# The Mushroom *Agaricus blazei* Murill Elicits Medicinal Effects on Tumor, Infection, Allergy, and Inflammation through Its Modulation of Innate Immunity and Amelioration of Th1/Th2 Imbalance and Inflammation

**DOI:** 10.1155/2011/157015

**Published:** 2011-09-06

**Authors:** Geir Hetland, Egil Johnson, Torstein Lyberg, Gunnar Kvalheim

**Affiliations:** ^1^Department of Cellular Therapy, The Norwegian Radium Hospital, Oslo University Hospital, Ullernchaussen 70, 0130 Oslo, Norway; ^2^Department of Gastroenterological Surgery, Ullevål Hospital, Oslo University Hospital, Oslo, Norway; ^3^The University of Oslo, Norway; ^4^Center of Clinical Research, Ullevål Hospital, Oslo University Hospital, Oslo, Norway

## Abstract

The medicinal mushroom *Agaricus blazei* Murill from the Brazilian rain forest has been used in traditional medicine and as health food for the prevention of a range of diseases, including infection, allergy, and cancer. Other scientists and we have examined whether there is scientific evidence behind such postulations. *Agaricus blazei* M is rich in the immunomodulating polysaccharides, **β**-glucans, and has been shown to have antitumor, anti-infection, and antiallergic/-asthmatic properties in mouse models, in addition to anti-inflammatory effects in inflammatory bowel disease patients. These effects are mediated through the mushroom's stimulation of innate immune cells, such as monocytes, NK cells, and dendritic cells, and the amelioration of a skewed Th1/Th2 balance and inflammation.

## 1. Introduction

**Figure 1:**
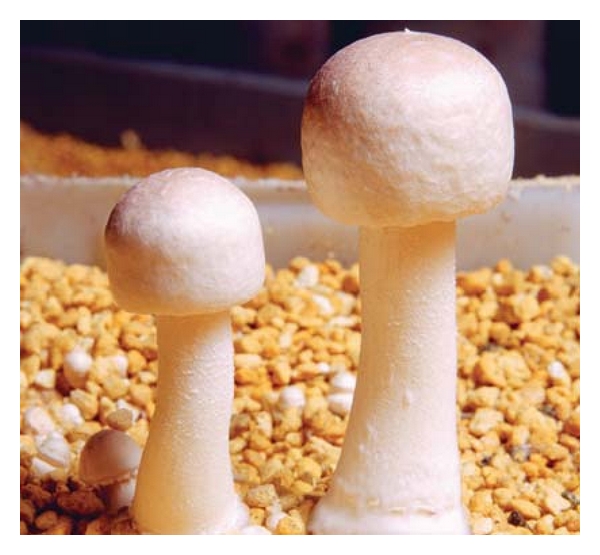
*Agaricus blazei* Murill. Photo NutriCon. The mushroom is cultivated commercially for the health food market in Japan, China, and Brazil. The AbM-based AndoSan extract is produced in Japan and developed and distributed by ImmunoPharma AS, Oslo, Norway.

The edible *Basidiomycetes* mushroom *Agaricus blazei* Murill (AbM), a.k.a. *Agaricus subrufescens* Peck (already described in 1893), and *Agaricus brasiliensis* Wasser [1] (Figure 1), of Brazilian rain forest origin is used in traditional medicine against cancer and various diseases [2, 3]. It is related to the champignon (*Agaricus bisporus*) and is shown to be rich in immunomodulating substances such as highly branched *β*-1,3-/1,6-glucans [4] and proteoglycans [5]. These are known ligands for CD11b/18 (complement receptor 3, CR3) [6], dectin-1 [7], and toll-like receptor 2 (TLR2) [8] on monocytes, dendritic cells (DC), granulocytes, and NK cells [9] of the innate immune system. AbM is also shown to contain agaritine and ergosterol (provitamin D2) that is found to induce apoptosis in leukemic cells [10] and inhibit tumor-induced angiogenesis [11], respectively, as well as isoflavonoids with potent hypoglycemic action that could be useful against diabetes mellitus [12]. AbM is reported to have antitumor properties in mouse models of fibrosarcoma, myeloma, ovarian, lung, and prostate cancer, and in human studies against gynecological cancer (increased NK cell activity and quality of life) and leukemia [13].

## 2. Effects on Infection and Allergy

**Figure 2:**
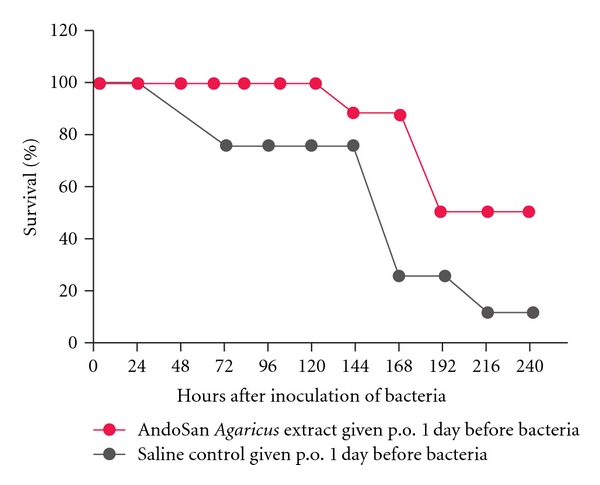
5-6-week-old female inbred NIH/Ola mice were given either 200 *μ*L of AndoSan AbM extract or phosphate-buffered saline (PBS) intragastrically a day before i.p. injection of 1 million colony-forming units of *Streptococcus pneumoniae* serotype 6B. There was a significant difference (*P* < 0.05) between survival after treatment with AndoSan (red line) and PBS (black line). From [14], permission granted for republication by Scand J Immunol, where the figure was originally published.

**Figure 3:**
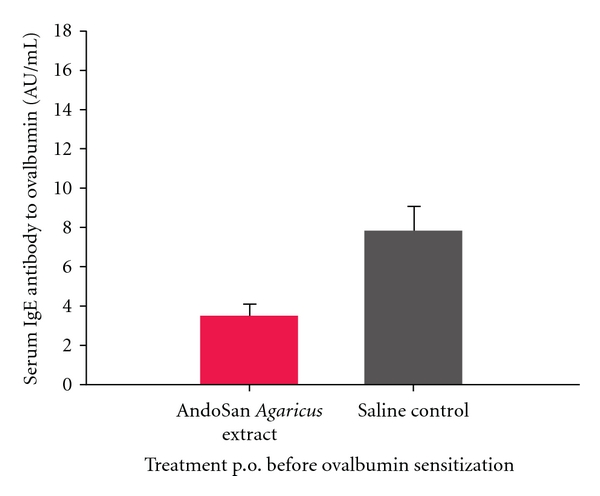
Female NIH/Ola mice were given either 200 *μ*l of the AndoSan AbM-based extract or PBS intragastrically on day 1 and 10 *μ*g of ovalbumin s.c. on day 0 and again on day 20, before exsanguination for serum on day 26. IgE antiovalbumin levels were lower in the AbM- than in the PBS-treated groups (*P* = 0.002). Similar results were found if AbM extract or PBS was given 3 weeks after the allergen immunization (not shown, please see [16]). IgG2a antiovalbumin levels (Th1 response) tended to show the opposite result (not shown). From [16], previously published by a BMC journal, which allows reuse.

We found that an AbM-based extract (AndoSan, http://www.immunopharma.net/), also containing the medicinal* Basidiomycetes* mushrooms *Hericium erinaceum* (15%) and *Grifola frondosa* (3%), given orally increased survival from bacterial sepsis in mice inoculated i.p. a day afterward with pneumococci (Figure 2) [14] or fecal bacteria [15]. The mixed mushroom extract also protected against IgE-mediated allergy in a mouse model when given p.o. either before or after ovalbumin s.c. sensitization of the animals (Figure 3) [16]. In supernatants of cultured spleen cells from the sacrificed AbM-treated mice, there was an increased T-helper cell 1 response relative to the allergy-inducing Th2 response. The observation fits with the reduced specific serum IgE levels in these animals and shows that also adaptive immunity is engaged by the mushroom. Since the original Th1/Th2 dichotomy [17] says that the antitumor and anti-infection Th1 response is inversely related to the Th2 response, the spleen cell finding above also helps explain the concomitant antiallergic, antitumor, and antiinfection effects of AbM. Moreover, this agrees with the very interesting report finding that AbM extract ameliorated a skewed Th1/Th2 balance both in asthma-induced and in tumor-bearing mice [18]. It is previously known that patients with advanced cancer have malfunctional Th1 cells and a Th2-skewed immune system [19]. However, it is not known whether AbM contributed to rectify a possibly induced Th1/Th2 imbalance in the above-mentioned sepsis models in mice [14, 15].

We have previously compared the biological potency of 5 different AbM products orally in a blinded experiment in the pneumococcal sepsis model and found that only AndoSan, given orally 24 h prior to bacterial challenge, induced statistically significant lower bacteremia and higher survival rate than did saline given prechallenge in control mice [13]. The outcome of this experiment, actually done in 2003 but not published until 2008, was the basis for choosing AndoSan (then called AbM extract A) in our further studies. Synergies between components from the three mushrooms in the said extract may explain its enhanced efficacy against sepsis. Tuberculosis is another serious infection although it actually only develops into active disease in 10% of those infected with *M. tuberculosis* bacilli. Hence, in contrast to the exposed but healthy individuals, the tuberculosis patients represent a selected group, which is not prone to the tubercle bacilli's strong ability to elicit Th1-type cellular immune responses, for example, the normal reaction to the BCG vaccine. In fact, the Th1/Th2 imbalance in these patients is shown by their higher frequency of allergy when compared with healthy controls [20, 21]. Although a *β*-glucan from yeast had a protective effect against *M. bovis*, BCG infection in a mouse model [22], the effect of AbM has not to our knowledge been examined in tuberculosis.

## 3. Anti-Inflammatory Effect

**Figure 4:**
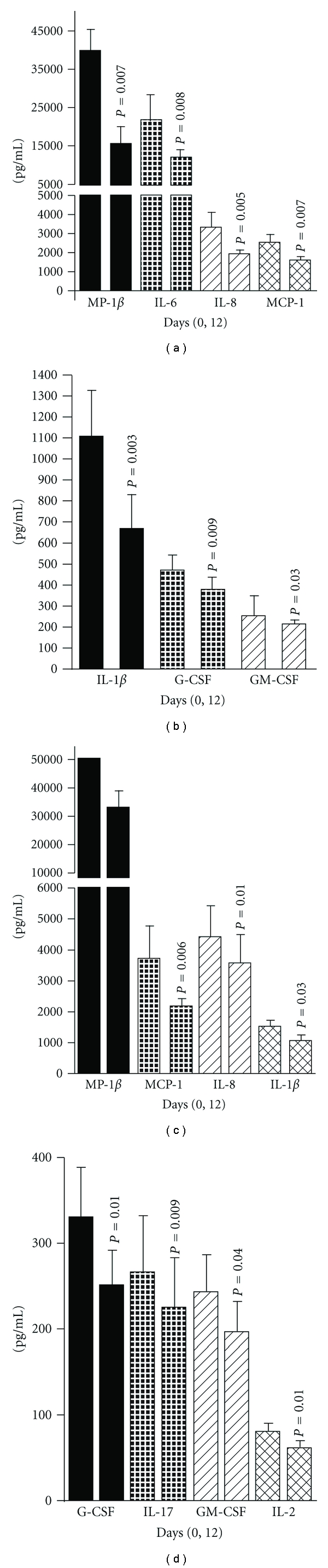
Bar graphs (mean and standard error the mean (SEM)) with levels of cytokines (pg/mL) MIP-1*β*, IL-6, IL-8, and MCP-1 (a) and IL-1*β*, G-CSF, and GM-CSF (b) in stimulated (LPS 1 ng/mL) whole blood *ex vivo* from eleven patients (unless otherwise stated) with ulcerative colitis prior to (day 0) and after AndoSan consumption for 12 days. Days 0 and 12 after stimulation are depicted by the first and second bars from the left, respectively. For MIP-1*β* and IL-8, measurements in nine out of ten patients were available. Corresponding measurements from eleven patients with Crohn's disease (unless otherwise stated) were significantly reduced for cytokines MIP-1*β*, MCP-1, IL-8, and IL-1*β* (c) and G-CSF, IL-17, GM-CSF, and IL-2 (d). For MIP-1*β* and IL-8, measurements in ten out of eleven patients were available. Despite remeasurement of MIP-1*β* after dilution of plasma 1/10, high out of range values still occurred. Accordingly, the true concentrations of MIP-1*β* were even higher. The *P* values between the bar graphs compare with the cytokine levels at day 0 prior to the intake of AndoSan. From [24] where the data was part of scatter plots in Scand J Immunol.

In a human phase I study in 15 healthy volunteers, we recently found no side effects after intake of the AbM-based AndoSan extract [23, 24]. In particular, there were no significant changes in general blood parameters and no negative effects on kidney, liver, or pancreas function. This agrees with a toxicity study over two years in rats [25], which rather found that animals ingesting the highest AbM concentration lived the longest, presumably due to reduced cancer development. In the mentioned phase I study, AbM extract had an anti-inflammatory effect as shown by significantly reduced levels of proinflammatory cytokines. Although this is contrary to our in vitro findings of increased production of proinflammatory cytokines by monocytes, monocyte-derived DC (MDDC), and human umbilical vein endothelial cells (HUVEC), we did, as a consequence of the phase I trial, conduct a clinical pilot study at our hospital on patients with the inflammatory bowel diseases, ulcerative colitis and Crohn's disease. The result was a significant decrease in plasma levels of proinflammatory cytokines after 12 days of AndoSan intake orally and also decreased levels of the inflammatory marker calprotectin in feces of ulcerative colitis patients [24].  Figure 4 shows that MIP-1*β*, IL-6, IL-8, MCP-1, IL-1*β*, G-CSF, and GM-CSF levels were reduced in the blood of ulcerative colitis patients and MIP-1*β*, MCP-1, IL-8, IL-1*β*, G-CSF, IL-17, GM-CSF, and IL-2 levels in blood of Crohn's patients, respectively. This anti-inflammatory property of AbM may also be of importance for the mushroom's therapeutic effect on allergy and asthma in mouse models [18, 21], both of which are inflammatory conditions, and it may bear promise for use against autoimmune diseases. In addition, it may explain some of AbM's antitumor effects discussed below.

## 4. Mechanism: Stimulation of Immune Cells

The reason for the forceful and swift engagement of innate immunity when encountering an edible and harmless mushroom, such as AbM, is its sharing of pathogen-associated molecular patterns (PAMP) with other highly poisonous species. Such mushrooms and fungi are usually a health threat due to the action of their toxins, for example, muscimol from *Amanita muscaria* and the vasoconstrictor ergotamine from *Claviceps purpurea*, or invasion in immunodeficient patients (e.g., *Aspergillus fumigatus*) or normal individuals (e.g., *Stachybotrys chartarum*).

PAMP, such as *β*-glucans, which form the main cell wall skeleton in mushrooms and fungi and are their signature molecule, are recognized immediately by so-called pattern-recognition receptors (PRR) [26], such as TLR2, dectin-1, and CR3. One can exploit this immune reaction by using an innocent mushroom such as *Agaricus blazei* M to enhance the body's protection against serious diseases. Although AbM induced NF-*κ*B activation via stimulation of TLR2 on cells in vitro [27], the AbM-based mushroom extract AndoSan had anti-inflammatory effect in inflammatory bowel disease patients in vivo [24]. In addition to monocytes, granulocytes, and DC, also NK cells bear such PRR [9] and are stimulated by AbM in vitro, for example, to induce increase in cytokine production, expression of the adhesion molecule CD11b on monocytes and granulocytes, and ROS and NO^−^ production in the latter cells [28]. An AbM extract also gave a dose-dependent increase in release of proinflammatory cytokines from HUVEC in culture [29]. Since human skin endothelial cells can express all 10 TLR genes [30], TLR-binding of AbM was probably one mechanism behind the above finding in HUVEC. This demonstrates that AbM also affects EC, which are important parttakers in the innate immune response. In a human study, AbM has been shown to increase NK cell activity in cancer patients [31]. Another important group of receptors on macrophages and other innate immune cells are cytosolic NOD (nucleotide-binding and oligomerization domain-) like receptors (NLR). These receptors detect conserved bacterial molecular signatures within the host cells, similar to recognition of *β*-glucans via surface receptors or so-called “danger signals,” alerting the immune system of hazardous environments [32], and “cross-talk” with TLR on DC [33]. 

It is also known that AbM can activate the alternative pathway of complement [34], giving binding of the CR3 ligand, iC3b, to particulate AbM and thus contribute to the AbM engagement of the phagocytic CR3. Moreover, the formation of complement activation split products and chemotaxins—C3a and C5a—when also the terminal complement pathway is activated will lead to their binding to C3a and C5a (CD88) receptors, respectively, and chemotaxis of the immune cells towards a C3a and C5a gradient and hence towards the AbM source. 

Animal studies have shown that although *β*-glucans can enter the proximal small intestine, the specific *β*-1,3-glucan backbone is indigestible [35]. In vivo, AbM most probably engage Peyer's patches in the intestines both via their abundant *β*-glucans and other immunomodulating substances such as proteoglycans, which may be transported to macrophages and DC after being taken up from the gastrointestinal lumen by M cells. Another possibility is direct uptake of or stimulation by such substances of DC via their processes into the gastrointestinal lumen or by intestinal macrophages that may fragment the glucans for transport to the bone marrow and reticuloendothelial system for further release and stimulation of other immune cells [35]. Previously, AbM has in fact been shown to stimulate endothelial cells in vitro [29]. Sorimachi et al. [36] demonstrated that ethanol (50%) precipitation of water extracts of the AbM fruiting body or its supernatant, rather than of the mycelium, induced TNF*α*, IL-8, and NO^−^ secretion in bone-marrow-derived rat macrophages. Since the complex *β*-1,3-/1,6-glucansfrom AbM induced different cellular activities than the mainly glucose-containing *β*-1,4-glucan from its nonmedicinal cousin *A. bisporus* (the champignon) [37], both differences in content, structure, and branching of their *β*-glucans must be highly important for the mushrooms' biological properties. Especially, *β*-1,3-glucans seem to be essential for immunological activity [37]. Interestingly, also a low-molecular-weight polysaccharide isolated from AbM has been reported to suppress tumor growth and angiogenesis in vivo [38]. Pyroglutamate is found to be another such small substance [39]. Since also the anti-allergic effect of AbM extract seems to be owing to low-molecular-weight substances (Hetland G, unpublished results) in a mouse model, other smaller and simpler substances than *β*-glucans in AbM could very well be as important for the mushroom's biological effects. 

Most probably the mushroom extract also affects the intestinal flora, which comprises 10 times more bacteria than cells in our entire body. Bacteria in the gut are known to produce essential vitamins and so forth, for example, bacteria that can ferment soy beans and produce K2 vitamin that is important for calcium metabolism [49]. It is probable that also intestinal bacteria either produce metabolites during digestion of AbM extract or produce analytes after themselves being stimulated by AbM. Such molecules may be biologically active after uptake from the gut and may affect the host.

The PAMP or NOD signature-bearing pathogens are annihilated immediately by the patrolling innate immune cells. Some of these like monocytes and DC—directors of the immune system orchestrating the linkage of innate and adaptive immunity—process antigens from the mushrooms/fungi and present them together with self HLA II molecules for CD4 T helper cells, thus engaging adaptive immunity against the intruders. 

**Figure 5:**
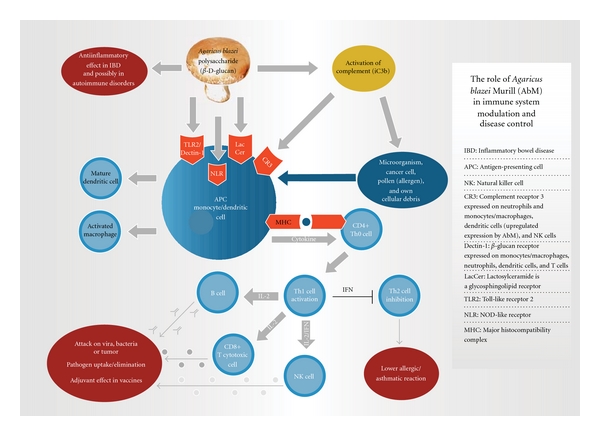
Role of the mushroom *Agaricus blazei* Murill in immune system modulation and disease control. It is assumed that besides *β*-glucans also other, yet unknown substances in the mushroom, probably of low molecular weight, do parttake in the action. IL-12 is the cytokine from the monocyte/dendritic cell that stimulates Th0.

Recently, we examined AbM-stimulated MDCC and found a significantly increased production of various cytokines [50]. This agrees with microarray studies in vitro of promonocytic THP-1 cells incubated with the AbM extract, which showed upregulation for most of genes associated with immune function, including the gene for IL-23*α* subunit—a Th1 cytokine in the IL-12 family [10]. In fact, the AbM extract induced even higher production of cytokines TNF*α*, IL-1*β* and MCP-1, and G-CSF than did cells stimulated with an appropriate concentration of 0.5 *μ*g/mL of LPS [50]. Hence, MDDC may in some respect be even more primed to defense against mushrooms/fungi than against Gram-negative bacteria. At our Department of Cellular Therapy, we construct autologous DC vaccines for clinical trials against various human cancers, by electroporating isolated cancer mRNA into dendritic cells harvested from the same patients. Benko et al. [33] have proposed the emerging of ligands of the innate recognition systems as new adjuvant candidates for vaccine design. In line with this school of thought, we are currently examining whether the above AbM extract may be used as immune adjuvant in such anti-cancer DC vaccines, similar to studies on DNA vaccines in the mouse against hepatitis B virus and mouth-and-foot diseases [51, 52]. In these vaccines, AbM was found to increase levels of antigen-specific antibodies as well as to promote proliferation of T cells, illustrating engagement of adaptive immunity.  Figure 5 shows a cartoon of the proposed role of AbM in immune system modulation and the resulting disease control. 

**Figure 6:**
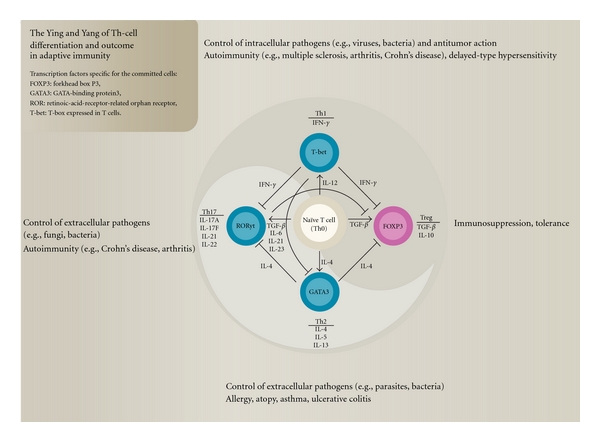
The Ying and Yang of adaptive immunity. There is a balance between the Th responses in such a way that Th1 inhibits Th2, which inhibits Th17 and which again inhibits the Treg response. “Over-shoot” of Th1 and Th17 responses in susceptible individuals can result in autoimmune disorders. The regulation by T reg cells results in immunosuppression and tolerance. The *Agaricus* mushroom shifts the Th1/Th2 balance towards increased Th1 response, which in addition to intracellular pathogens also fights cancer.

In Figure 6, the balance between the different Th responses is depicted as the Ying and Yang of adaptive immunity. The committed cells are characterized by expression of the specific transcription factors; T-bet for Th1, GATA-3 for Th2, FoxP3 for Tregs, and ROR*γ*t for Th17 cells [53]. The cartoon shows that whereas both Th1 and Th2, cells inhibit Th17 and Treg cells, Th1 inhibits Th2, and Th17 inhibits Treg cells. It is noteworthy that in Crohn's disease, which is proposed to be a Th1/Th17-type autoimmune disease, we found significant reduction in plasma levels of IL-2 (Th1 cytokine) and IL-17 after intake of the AbM-based AndoSan extract by the patients [24]. In the other patients with ulcerative colitis, which is proposed to have a Th2-type autoimmune pathogenesis, we found that the IL-4 and IL-13 levels tended to decrease, although not statistically significantly, after AndoSan's intake [24].

## 5. Antitumor Effects and Proposed Mechanism behind

As mentioned, the Agaricus mushroom is reported to inhibit various tumors [10], including hematological cancers such as myeloma in a recent mouse model [43] and leukemia in a human study [42]. One proposed mechanism behind the antitumor effects of AbM is the induction of apoptosis in tumor cells, which is demonstrated in vitro [40]. This is confirmed by the microarray finding of increased expression of genes inducing apoptosis as well as inhibition of cell division [46] in PBMC from patients with hepatitis C virus infection who drank AbM extract for 1 week. Other contributing mechanisms are (i) the known antitumor action of ergosterol [11, 54] contained in AbM extract, (ii) the anti-inflammatory effect of AbM [23, 24], which may reduce levels of the “tumor-friendly” neoangiogenic and granulocyte-chemoattractant factor IL-8 and thus may also decrease intratumor formation of reactive nitrogen and oxygen species that may hamper the infiltration of cytotoxic T cells [55], and (iv) the amelioration of the proposed skewed Th1/Th2 balance in advanced cancer [18]. There is a well-established connection between inflammation and tumorigenesis [56], and it is known that organs with chronic inflammation are prone to cancer, for example, the colon in inflammatory bowel diseases, the pancreas after chronic pancreatitis, and the liver secondary to chronic viral hepatitis. Since up to 1/4 of all cancers are estimated to be caused by underlying infections and inflammation [57], there has lately been a novel interest for the use of anti-inflammatory treatment in cancer therapy [45]. Hence, both the anti-inflammatory [23, 24] and anti-infection [14, 15] properties of AbM that we have disclosed may contribute to the mushroom's antitumor activity. Interestingly, whereas there was a little, nonsignificant reduction in HCV load in serum in the mentioned clinical pilot trial in 4 patients with chronic IFN*α*-resistant HCV infection, the gene for IFN*αβ* receptor was significantly upregulated by the 7 days AbM treatment [46].

Currently at our hematological department, we are conducting a placebo-controlled, double-blinded phase II trial in patients with multiple myeloma, who have been drinking the AbM-based AndoSan extract as a supplementary treatment. These patients are subjected to standard high-dose chemotherapy and autologous hematopoietic stem cell transplantation after the harvesting of the cells at our Department of Cellular Therapy. Patients with multiple myeloma were chosen for such a clinical trial because there is no curative therapy for this cancer today-only life-extending treatment. In the other known clinical studies with AbM, the mushroom was reported to have positive effect against nonlymphocytic leukemia [42] and to increase quality of life and NK-cell activity in blood of gynaecological cancer patients on high-dose chemotherapy [31]. In a small AbM study on prostate cancer in patients enrolled after radical prostatectomy, there was no reduction in their prostate-specific antigen (PSA) levels [58]. However, there were no placebo controls, and the study included many with PSA values below the guideline of 0.2 ng/mL of PSA. That study is in contrast with the findings of AbM-induced apoptosis for prostate cancer cells and inhibited tumor growth and antiangiogenic effect in a mouse model [44]. Interestingly, a recent paper reported evidence for suppressed growth and invasiveness of human breast cancer cells in vitro after treatment with a blend of AbM and other medicinal mushrooms [59].  Table 1 gives an overview over the reported in vivo antitumor effects of the *Agaricus* bM mushroom, and Table 2 shows clinical studies with this mushroom.

## 6. Conclusions

The medicinal mushroom *Agaricus* bM has been shown to have beneficial effects on a range of diseases including cancer, infections, allergy/asthma, and inflammatory disorders. The explanation is the mushroom's engagement of innate immunity, which is “broad-spectered.” When adaptive immunity then is engaged through the stimulation of DC, it results in an enhanced Th1 antitumor and anti-infection response relative to the proallergic Th2 response. Thus, AbM ameliorates the Th2-skewed balance found in advanced cancer, mycobacterial infections and allergy and asthma. In addition, there may be a general anti-inflammatory effect of AbM, which may be therapeutical for inflammatory bowel diseases and augment the mushroom's antitumor and antiallergy/antiasthma properties. Hence, AbM extract may show promise as a prophylacticum and as an additive treatment for quite different and some serious diseases. 

## Figures and Tables

**Table 1:** Reported in vivo antitumor effect of the mushroom *Agaricus blazei* Murill.

Tumor/related activity	In vivo effects of *Agaricus* bM	Refs. (no., year, country)
Fibrosarcoma	Inhibition of tumor size and neoangiogenesis	[4] 1994, [40] 1998, [3] 2001, [39] 2004, Japan
Gynecological cancer*	Increased NK-cell activity and increased quality of life (QOL)	[31] 2004, Republic of Korea
Ovarian cancer	Inhibition of metastasis	[41], 2005 Japan
Lung cancer	Inhibition of metastasis	[39] 2004, [41] 2005, Japan
Leukemia*	Inhibition	[42] 1994, Japan
Myeloma	Inhibition of tumor size	[43] 2007, Japan
Prostate cancer	Reduced tumor growth and neoangiogenesis No significant effect on PSA values*	[44] 2009, Taiwan [45] 2010, Japan
Carcinogenicity	No	[25] 2008, Republic of Korea

*Human studies, otherwise studies in mice.

**Table 2:** Clinical studies with *Agaricus blazei* Murill (AbM).

Patients disease	No. of subjects	Treatment	Clinical effect	Refs. (no., year, country)
Acute nonlymphocytic leukemia	10	Chemo + AbM	Inhibition of leukemic cells	[42], 1994, Japan
Gynecological cancer	100	Chemo + AbM Kyowa or placebo, 9 wk	↑ NK cell activity, ↑ QOL	[31], 2004, Republic of Korea
IFN*α*-resistant chron. HCV infection	4	AndoSan extract 60 mL/d for 7 d	Insignificant ↓ HCV load, but ↑ IFN*αβ* receptor and “antitumor gene” expression	[46], 2006, Norway
Chron. HBV infection	4	AbM extract 1.5 g/d, 12 mo	Normalized liver function; ↓ASAT ↓ ALAT levels	[47], 2007, Taiwan
Diabetes type 2	72	AbM 1.5 g/d or placebo 12 wk	Improved insulin resistance	[48], 2008, Taiwan
Healthy volunteers	15	AndoSan 60 mL/d for 12 d	No toxicity, Anti-inflammatory	[23], 2009, Norway
Inflammatory bowel diseases (*Ulcerative colitis and Mb Crohn)	21	AndoSan 60 mL/d for 12 d	Anti-inflammatory; ↓ proinflammatory cytokines in blood and ↓*calprotectin in feces	[24], 2011, Norway

Abbreviations: ASAT aspartate aminotransferase, ALAT alanine aminotransferase, QOL quality of life. AndoSan is an AbM-based extract also containing 15% *H. erinaceum* and 3% *G. frondosa.*
